# Comparing a new visuospatial intervention administered 3 days after a trauma film to reduce the occurrence of intrusive visual memories: a single-center randomized, controlled trial in healthy participants

**DOI:** 10.3389/fpsyg.2024.1454086

**Published:** 2025-01-10

**Authors:** Jan-Martin Matura, Henrik Kessler, Emily A. Holmes, Nina Timmesfeld, Marianne C. Tokic, Nikolai Axmacher, Simon E. Blackwell, Anna-Christine Schmidt, Johanna M. Schweer, Charlotte Hippert, Lukas Apel, Jan Dieris-Hirche, Stephan Herpertz, Aram Kehyayan

**Affiliations:** ^1^Department of Psychosomatic Medicine and Psychotherapy, LWL-University Hospital, Ruhr-Universität Bochum, Bochum, Germany; ^2^Department of Psychosomatic Medicine and Psychotherapy, Fulda Hospital, University Medicine Marburg Campus Fulda, Fulda, Germany; ^3^Department of Women’s and Children’s Health, Uppsala University, Uppsala, Sweden; ^4^Department of Medical Informatics, Biometry and Epidemiology, Ruhr-Universität Bochum, Bochum, Germany; ^5^Department of Neuropsychology, Faculty of Psychology, Institute of Cognitive Neuroscience, Ruhr-Universität Bochum, Bochum, Germany; ^6^Department of Clinical Psychology and Experimental Psychopathology, Institute of Psychology, University of Göttingen, Göttingen, Germany

**Keywords:** visuospatial task, intrusive memories, posttraumatic stress disorder, PTSD, *Mobilum*, *Tetris*, trauma film

## Abstract

**Introduction:**

Intrusive memories occur frequently after potentially traumatic events and form a core symptom of posttraumatic stress disorder (PTSD) if they persist. The translational approach of visuospatial interventions tries to target those intrusive memories in order to reduce their frequency predominantly using an intervention including as one component the computer game *Tetris.* Despite promising results, the application of *Tetris* has critical drawbacks, e.g., potential commercial or copyright issues. Furthermore, it remains unclear whether it is this specific game or, as predicted by theory, a visuospatial task *per se* that leads to the effect. This study hence aims to compare the effect of *Tetris* with an alternative, bespoke visuospatial task: *Mobilum* developed for the current purpose.

**Methods:**

*N* = 120 healthy participants watched a trauma film and recorded their intrusive memories in a diary for 6 days. Three days after watching the film, they were randomized to 3 groups and after memory reactivation cue received either *Tetris* or *Mobilum* or *Control* (no task). Prior to intervention 8 participants reported zero intrusive memories to the film and were excluded from further analyses, therefore 112 participants were included in the analysis.

**Results:**

A mixed Poisson regression model revealed that the *Mobilum* group had significantly less frequent intrusive memories after the intervention compared to the control condition (approximately 43%, *p* = 0.0013). There was no significant difference for the *Tetris* group compared to *Control* (17% less frequent, *p* = 0.3798).

**Discussion:**

Our results suggest that visuospatial tasks other than *Tetris*—in this case, *Mobilum*—can also lead to a reduction in intrusive memories when administered 3 days after a trauma film. This strengthens the assumption that it is not specifically the game *Tetris*, but rather the visuospatial nature of the task, that is responsible for the reduction. Aspects of further investigating the potential of *Mobilum* as well as clinical implications are discussed.

## 1 Introduction

Many people experience potentially traumatic events in their lifetime. Some of them eventually develop trauma-related disorders including, but not limited to, posttraumatic stress disorder (PTSD), a disorder with a high world-wide prevalence (World Health Organization, 2013; Atwoli et al., 2015). There is evidence for the effectiveness of various forms of psychotherapy for the treatment of PTSD (Bisson et al., 2013) and many international guidelines recommend psychotherapy as a first-line treatment (National Institute for Health and Care Excellence, 2018; Schäfer et al., 2019; American Psychological Association, 2017). Unfortunately, on a global scale, only a small minority of patients actually receives any form of treatment for PTSD, causing enormous suffering and societal costs (Kessler, 2000; Bisson et al., 2013). The need to establish new treatments to effectively tackle PTSD symptoms on a larger scale could be met by two converging approaches: The digitalization of treatment methods, and the translation of experimental research to clinical practice. The former would increase availability, while the latter could increase efficacy by directly addressing key PTSD symptoms (translational medicine). The overarching aim of our study presented here follows the call by Holmes et al. (2014) to develop new science-based psychological interventions (in this case tackling PTSD).

In this vein, there have been approaches to develop new treatment forms that apply visuospatial tasks to reduce the frequency of one prominent symptom of trauma-related disorders: visual intrusive memories of traumatic events (Kessler et al., 2020; Iyadurai et al., 2019; Astill Wright et al., 2021). Such intrusions are typically visual mental images from traumatic events (e.g., appearing as still pictures or brief film clips) that occur involuntarily, are hard to control, and cause suffering (Brewin, 2001; Ehlers and Clark, 2000; Iyadurai et al., 2019). Two concepts guide the use of visuospatial tasks to mend intrusions after traumatic experiences: dual-task interference and memory reconsolidation-update accounts. Mental imagery, the cognitive substrate of intrusions, involves visuospatial working memory, which has a limited processing capacity (“bottleneck”). A demanding visuospatial task applied concurrently while holding an intrusive image in mind (dual-task) interferes with intrusion-related imagery competing for the same limited resources. This typically decreases emotionality and vividness of the mental image (Baddeley and Andrade, 2000; Andrade et al., 1997; van den Hout et al., 2001; Engelhard et al., 2010).

The second concept, memory reconsolidation-updating, suggests that memories that have already been consolidated eventually become amenable again, if they are reactivated and afterwards subjected to interference (Misanin et al., 1968; Nader et al., 2000). This insight from cognitive neuroscience opens up the possibility that old traumatic memories can still be changed (updated) when they are reactivated while another task interferes with the reconsolidation process (Monfils and Holmes, 2018; Astill Wright et al., 2021).

One promising cognitive task with the potential to reduce the frequency of visual intrusions as part of an imagery-competing task procedure, incorporates the popular and visuospatially demanding computer game *Tetris*. In *Tetris*, the game principle is to create continuous lines out of different blocks moving from the top to the bottom of the screen by rotating those blocks. In several experimental studies testing this novel intervention, healthy participants watched a trauma film with aversive material and played *Tetris* during or after the film (in that latter case following a memory cue), which significantly reduced the number of visual intrusions of the film during the following days (Holmes et al., 2009; Holmes et al., 2010; Deeprose et al., 2012; Lau-Zhu et al., 2017). In clinical applications it has also been shown that the imagery competing task procedure including *Tetris* gameplay can reduce intrusions when played within a time interval of 6 h following a road traffic accident (Iyadurai et al., 2018) or traumatic childbirth (Horsch et al., 2017). Adding the concept of reconsolidation-updating, the time interval between “traumatic event” (trauma film in experimental research or actual trauma in patient populations) and the visuospatial intervention has been expanded substantially in recent studies. The novel intervention has been investigated to reduce intrusion frequency in the following days even if applied 24 h (James et al., 2015) or 72 h (Hagenaars et al., 2017; Kessler et al., 2020) after watching the trauma film in healthy participants. In a study of long standing memories of trauma, we let PTSD patients write down the content of an intrusive memory (in many cases decades old) and play *Tetris* afterwards. In the following weeks they experienced a greater reduction of intrusions of exactly that scene they had targeted with the novel intervention (in relation to the intrusion frequency in the weeks before the intervention), compared to the reduction of other intrusive memories of other traumatic scenes, that were monitored, but not targeted by the intervention (for these, reduction was measured as intrusion frequency in the second versus the first half of the inpatient treatment period) (Kessler et al., 2018). Other studies in clinical populations suffering from trauma-related symptoms include a single case series with refugees in Sweden (Kanstrup et al., 2021), or studies with intensive care workers suffering from intrusive memories of work-related trauma during the COVID-19 pandemic (Iyadurai et al., 2023; Ramineni et al., 2023).

While by now, *Tetris*-based interventions have shown to be effective in several studies using different study designs, based on the theoretical assumptions, other visuospatial tasks that could elicit dual-task interference and interfere with reconsolidation should lead to comparable results. To better understand the mechanisms behind the effects observed in these *Tetris*-based interventions, it is necessary to test *Tetris* against other tasks, with which it shares common features (in this case: being visuospatially demanding).

The study presented here critically extends previous laboratory research by introducing an alternative visuospatial task, *Mobilum* (Kessler et al., 2019), and testing it alongside *Tetris* in a design that is parallel to the one published in 2020, i.e., 3 days after trauma film viewing (Kessler et al., 2020). In *Mobilum*, participants solve three-dimensional tasks by mentally “rotating” a virtual cube to decide from which perspective an enclosed geometrical object is seen (see methods for details).

In our lab, we developed our own computer game *Mobilum* to be tested in future studies as an alternative to *Tetris* for the following reasons: (1) If *Mobilum* had a comparable effect on intrusions as *Tetris*, this would be an important step in building convergent evidence for the assumption based on theory that it is a visuospatial task *per se*, rather than the specific game *Tetris*, that could interfere with intrusive memory processing. *Mobilum* is designed to strongly engage the user in visuospatial processing, while minimizing other, potentially interfering factors present in *Tetris*, such as importance of reaction speed under time pressure, or colorful animations and attention-grabbing background designs. (2) Using a custom-made game enables researchers to fully control many variables as potential factors influencing the intervention effect: duration of game play, difficulty levels, visual appearance, and others. (3) *Tetris* is a licensed game. Therefore, commercial interests could counteract the idea of an intervention that is free for researchers, clinicians and patients.

This study aimed to test, whether *Mobilum* could lead to a reduction in intrusion rates comparable to the effect shown for *Tetris* in previous studies. To this end, 3 days after watching the trauma film participants were randomly allocated into 3 groups: they would either play *Mobilum*, *Tetris*, or perform no task (*Control*). Afterwards, they recorded the occurrence of intrusions for 3 more days in an intrusion diary. The main hypothesis was that participants in both the *Tetris* and the *Mobilum* conditions would have lower intrusion rates compared to the *Control* group after the intervention. If both of the two interventions were to show an effect, we planned an exploratory analysis to compare *Tetris* and *Mobilum* directly, to see if one might have a stronger impact than the other (but having no *a priori* hypothesis that this would be the case). Building on our previous observations of the development of intrusion frequency over the days (see Figure 3 in Kessler et al., 2020), we used count-based data and analyzed intrusion rate rather than means of intrusion numbers as the main outcome.

## 2 Materials and methods

### 2.1 Participants

Based on average estimated effect sizes obtained in earlier studies with the Tetris intervention (Kessler et al., 2020; Holmes et al., 2010; James et al., 2015), we chose to investigate a sample size of 120 participants. Potential participants were screened via online questionnaires. Exclusion criteria met by potential participants were: current or completed psychotherapeutic treatment (*N* = 7); five or more potential traumatic events assessed by the Essen Trauma Inventory (*N* = 0) (ETI; Tagay et al., 2006); Global severity Index (GSI) ≥ 0.58 measured with the Symptom Checklist-90-Revised (*N* = 11) (SCL-90-R; Franke, 1995); presence or suspicion of posttraumatic stress disorder (PTSD) represented by a sum score of ≥33 in the PTSD Checklist for DMS-5 (*N* = 0) (PCL-5; Weathers et al., 2013); participation in an experimental study using visuospatial intervention in the past (*N* = 2). After screening, a total of *N* = 120 non-clinical participants were recruited from four university campuses and a school of nursing. All participants provided their informed consent in written form.

Participants who experienced zero (0) intrusions during the 72 h following the trauma film were excluded from further analysis (3 from the *Mobilum* group, 5 from the *Control* group), since the study is predicated on the presence of intrusions during the first 3 days. Thus of 120 recruited, 112 participants were included in the analysis. The study was approved by the ethics committee of the faculty of psychology of the Ruhr-University Bochum, Germany (Nr. 273).

### 2.2 Procedure

On Day 1, all participants were asked to practice playing both the computer game *Tetris* and *Mobilum* for 5 min, to know how the games work, get some practice in the tasks and some basic knowledge of mental rotation regardless of which experimental condition they were subsequently randomly allocated to. After game practice, they received specific instructions in how to view the trauma film (e.g., to get emotionally involved, as if they were there as a bystander, and not to look away from the screen; following Kessler et al., 2020). Then they watched the trauma film for approximately 15 min sitting alone in a darkened room. Participants were next given detailed instructions on a definition of intrusive memories: *involuntary* memories of the trauma film that are hard to control and have typically the form of a mental image (from brief “pictures” to longer “films”); on the other hand, *intentional* thinking of the trauma film did not count as an intrusive memory even when it includes or is followed by mental images. They were then instructed how to use the intrusion diary for recording any intrusive memories from the film they would experience over the following 72 h (Holmes et al., 2010; James et al., 2015; Kessler et al., 2020).

Prior to their second laboratory session on Day 4, 72 h after presentation of the trauma film, participants were randomly allocated to group (*Control, Tetris*, or *Mobilum*) using a minimization scheme, see below (Scott et al., 2002; Altman and Bland, 2005). All groups were given the memory reminder task (watching non-traumatic stills of the trauma film and recalling the scene they belonged to) as reported in Kessler et al. (2020). There was then a 10 min break (as in Kessler et al., 2020; James et al., 2015)—the interval was standardized by containing a music filler task, where participants had to rate the pleasantness of pieces of music played for them.

**Figure 1:**
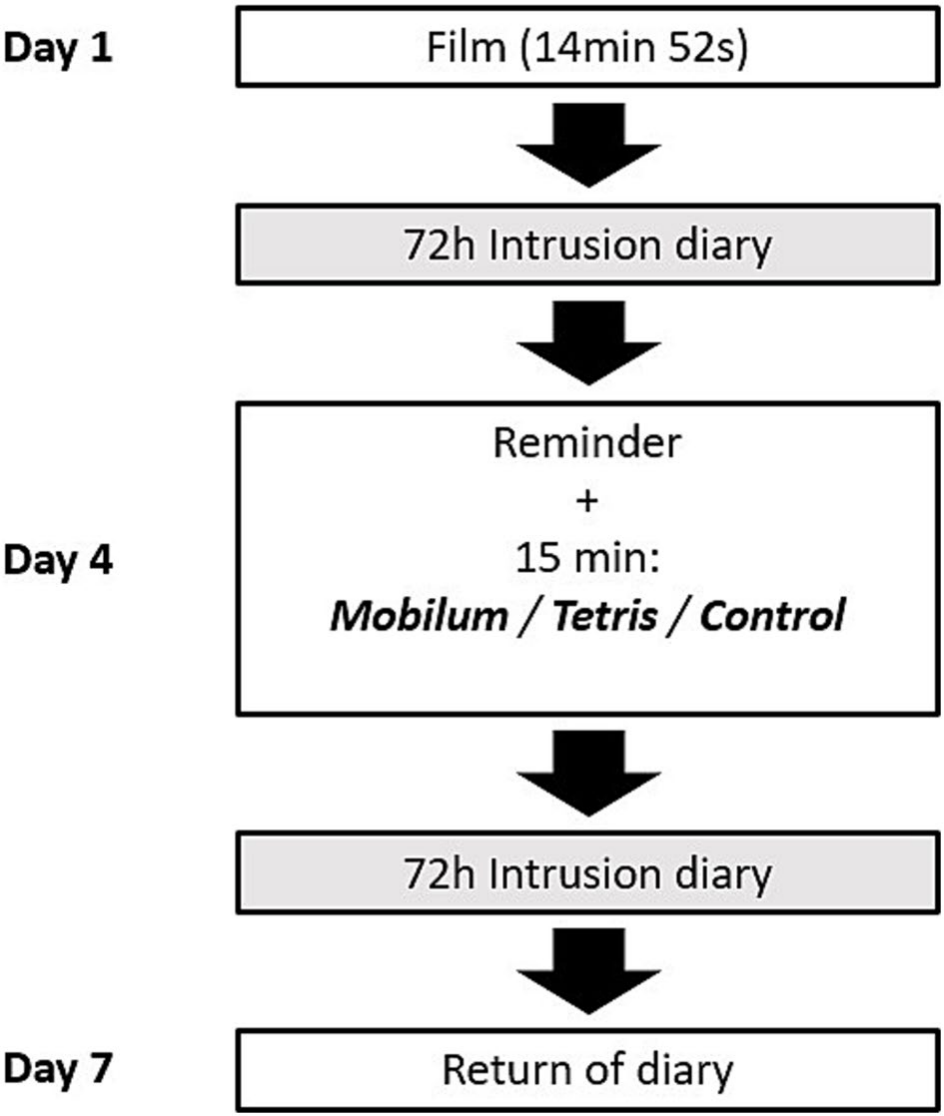
General study design. The testing took place over the course of 7 Days, starting with the first laboratory session (referred to as Day 1) and a chronological interval of 72 h between the following two laboratory sessions. On the first session, the participants viewed a trauma film, followed by 72 h during which they recorded all the film-related intrusive memories in the intrusion diary. On the second session (Day 4) they completed a reminder task of the trauma film and were randomly allocated to one of three following between-subject conditions: reminder-plus-Mobilum (Mobilum), reminder-plus-Tetris (Tetris) or reminder-only (Control). Following this session, they were asked to record their intrusions for another 72 h followed by the last session (Day 7) to hand over the diary.

After this memory reminder and 10 min break, participants either played *Tetris*, *Mobilum*, or they sat quietly in the *Control* condition for 15 min, each with the experimenter present. All participants were then reminded of the instructions for keeping the diary and were asked to record their intrusions in the diary for further 72 h. After these 72 h, during the last laboratory session (on Day 7), participants were asked to hand over the completed diary. An overview of the study design is shown in Figure 1.

### 2.3 Trauma film

The 14 min 52 s long trauma film was composed of 16 different scenes with material with traumatic content, i.e., events of exposure to threatened or actual death, or serious injury to others (American Psychiatric Association, 2013), as well as highly aversive scenes involving animals. The scenes contained car accidents, violent scenes or surgical procedures. The film used in the present study is analog to the film used in Kessler et al. (2020) (for more details, see Kessler et al., 2020, Supplementary Table S3). The film was watched in a dark room using a 17-inch laptop computer (comparable to Kessler et al., 2020). The viewing distance was approximately 15 inches. The instruction was paying close attention to the film. Furthermore, participants should try to imagine themselves as a bystander at the scene.

### 2.4 Intrusion diary

Participants recorded all intrusive memories related to the trauma film in a pen and paper diary during the 6 days of the study (day 1 to 3 = 72 h: pre-intervention, days 4 to 6 = 72 h: post-intervention). The diary has already been used in similar form in, e.g., Kessler et al. (2020) (see also James et al., 2015). In the diary, each day was indicated by a single printed box. This box had three sections for morning, afternoon and evening. Participants marked the correct section for each time an intrusive memory has occurred. They should ideally record all intrusions directly after occurrence, but at least check daily that the diary was maintained. They could also indicate that they had not at all experienced any intrusive memory in a particular section. The diary was explained verbally and written instructions were provided. The definition of intrusive memories was the same as in Kessler et al. (2020). Any involuntary memory of the film was classified as intrusion. In this vein, participants should not include deliberately recalled memories. In addition, the instruction stated that intrusive memories had to be in the form of “mental images” (e.g., a picture in your mind’s eye), and not of verbal thoughts.

Importantly, as in Kessler et al. (2020), days 1–6 were divided in 24 h intervals rather than calendar days (as in other studies using similar set-ups, e.g., James et al., 2015; Hagenaars et al., 2017). That means, day 1 was the first 24 h interval after the first study appointment (e.g., if this appointment ended at 3 pm, it would span from 3 pm that day to 3 pm the next day), day 2 the second 24 h interval, and so on. Day 4 was the 24 h interval immediately following the second study appointment, and the start of the post-intervention time period on day 4 was marked clearly in the diary to make sure that days 1–3 and days 4–6 were unambiguously separated as pre-intervention and post-intervention for all participants.

For data quality reasons, participants rated the diary compliance at 2 time points. Ratings were assessed for the first 3 days of the diary at the second laboratory session (pre intervention) and for the following 3 days at the third laboratory session (post intervention). The participants should rate to which percentage (range from 0 to 100%) they had recorded intrusions in the diary. For example, a participant who had 10 intrusions during the first 3 days of the study but had recorded only 9 of them would rate 90% for the diary compliance relating to the first 3 days of the study.

### 2.5 Tasks

#### 2.5.1 Memory reminder

In accordance with Kessler et al. (2020), the memory reminder procedure was split in two parts: (i) cue presentation followed by (ii) a music filler for 10 min:

i) The cue presentation procedure had three steps. First, for each scene (viewed 72 h previously) two visual images were presented simultaneously using PowerPoint. For each scene there were 2 images, hence there were 32 images overall. All images showed a moment briefly before the traumatic content, which means they were *not* picturing the worst moments themselves. Participants should recognize the film scene and indicate that by pressing a button. Then, the second slide instructed to recall the film scene as vividly as possible with eyes closed. In a third step, participants saw a pause monitor and could continue to the next pair of images by pressing the button again. Thus, participants could control the duration of presenting the slides.
ii) After presentation of all reminder cues, a time interval of 10 min took place to initiate the reconsolidation process. The amount of time was based on reconsolidation studies in animals (Nader et al., 2000) and humans (Schiller et al., 2010; Agren et al., 2012). We used a music filler task. Participants listened to music and rated the pleasantness of it afterwards (as used in James et al., 2015, Kessler et al., 2020).

#### 2.5.2 *Mobilum* computer game

Professional game programmers developed *Mobilum* (Kessler et al., 2019) with design and gameplay input from the research team bespoke for the purpose of the current study—i.e., to maximally interfere with image based memories after trauma. The application uses the Unity 5 game engine (https://unity3d.com/de/unity) and is implemented in the programming language C#. *Mobilum* is provided free of cost in the Google Play Store (working title: “Atomium,” https://play.google.com/store/apps/details?id=com.ViMaSter.Atomium) and runs on Android devices. Participants have to solve three-dimensional tasks and are considered to be deeply engaged in mental rotation. Basically, they have to imaginatively “rotate” a virtual cube in order to decide from which perspective a complex geometrical figure is seen. Within 15 min they are instructed to make as few mistakes as possible (precision, minus score for every wrong answer) and complete as many tasks as possible (speed, plus score for every correct answer). In principle, the game design shows two identical objects from different perspectives, so they appear differently on the first sight. Left sided, a transparent cube is displayed with a complex three-dimensional figure inside. This figure consists of more or less colored geometric objects (e.g., spheres, pyramids) connected by colored lines. This cube is by definition always shown from the front. Right sided, the same cube is viewed from another perspective. The five other possible perspectives (left and right, top and bottom, as well as back) are displayed on the bottom of the screen. Therefore, a cube with the respective perspective highlighted is shown for each option. Participants have to choose the right answer by touching the appropriate cube. Immediately after the user’s decision, the correct answer is shown, and the right cube slowly rotates to demonstrate the correct solution. The difficulty level is adaptive with more complex figures emerging after successive correct answers and less complex figures after successive wrong answers. This is to create a challenging but not frustrating gameplay experience. A timer counts down from 15 min until it reaches zero leading to a game stop and final score. A 10.1 inch Samsung Galaxy Tab 2 was used in this study. The game play duration, difficulty and appearance of the figures can be adjusted to suit different research use-cases.

**Figure 2:**
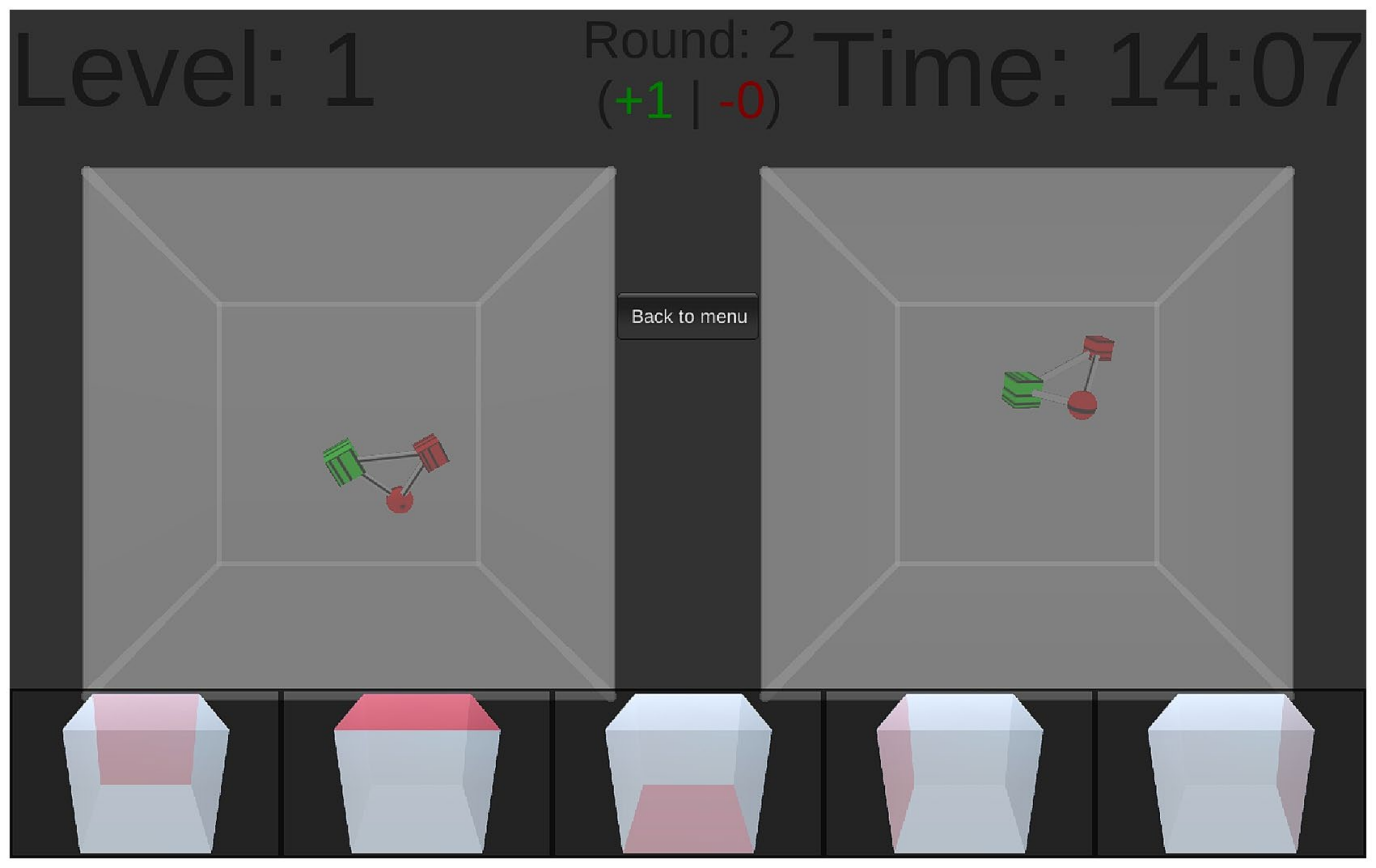
Screenshot of the game. On the left side, a transparent cube is displayed with a complex three-dimensional figure inside. This cube is by definition always shown from the front. On the right side, the same cube is viewed from another perspective. All five possible other perspectives (left and right, top and bottom, as well as back) are displayed on the bottom of the screen. The task is to identify from which of these perspectives the left cube has to be viewed to result in the right cube by touching the appropriate cube on the bottom of the screen.

See Figure 2 for a screenshot of the game.

#### 2.5.3 *Tetris* computer game

For this study, the mobile version of *Tetris* created by Electronic Arts (EA Mobile Montreal Team, 2014), version 2.2.07, set to ‘Marathon’ mode was used on a 10.1 inch Samsung Galaxy Tab 2 for a duration of 15 min. In *Tetris*, different kinds of objects must be arranged correctly. There are seven differently formed and colored blocks. These blocks are moving from the top to the bottom of the screen once at a time. The blocks can be rotated, while moving over the screen by touching the tablet screen accordingly. When a complete horizontal line of blocks results at the bottom of the screen, the line vanishes and the player is rewarded with points. Incomplete lines fill the screen step by step arising from the bottom of the screen. When they touch the top of the screen, the game ends. Participants were instructed to use their ‘mind’s eye’ to calculate the optimal position for the blocks to create horizontal lines to be awarded with points, as in Kessler et al. (2020) and James et al. (2015).

#### 2.5.4 Control task

For a duration of 15 min, participants in the control group sat quietly in the laboratory and could think about anything they would like. Other activities, such as using mobile devices, were strictly forbidden.

### 2.6 Pre-laboratory screening

#### Procedure

Prior to the first study appointment participants received access to questionnaire measures via a standardized email. These questionnaires screened for inclusion eligibility.

#### 2.6.1 Essen trauma–inventory

Potential traumatic events in participants’ history were assessed using the ETI trauma list (first part of the ETI questionnaire; Tagay et al., 2006). The list provides 15 different traumatic events. A cut-off of 5 events was set to exclude participants from the study. This was based on our experiences with another study with healthy participants (Kessler et al., 2020).

#### 2.6.2 Symptom checklist-90-revised

The SCL-90-R measures psychological symptoms and distress. This self-report symptom inventory consists of nine symptom dimensions with 90 items overall. There are 3 summary global scores, namely the Global Severity Index (GSI), the Positive Symptom Distress Index (PSDI) and the Positive Symptom Total (PST). In this study, the GSI assesses participants’ distress levels and a cut-off of ≥0.58 for exclusion was set. For the GSI, internal consistency is reported between 0.96 and 0.98. Test–retest reliability for GSI is *r* = 0.90 (Franke, 1995).

#### 2.6.3 PTSD checklist for DSM-5

The PCL-5 (Weathers et al., 2013) is a well-established questionnaire and can be used for diagnosis or development measure of posttraumatic stress disorder. Twenty items are spanning the four symptom clusters of PTSD according to DSM-5 (intrusion, avoidance, negative alterations in cognition and mood, alterations in reactivity and arousal). Each item can be rated between 0 (“not at all”) and 4 (“extremely”). Thus, the overall score can lie between 0 and 80. Scores of ≥33 suggest the diagnosis of PTSD and were therefore classified as exclusion criteria (Krüger-Gottschalk et al., 2017).

### 2.7 Data analysis and statistics

#### 2.7.1 Random allocation to groups

For group allocation, a minimization scheme was used (Scott et al., 2002; Altman and Bland, 2005). Based on previous work, randomized group allocation was dependent on number of intrusions experienced during the baseline phase ( ≤9vs>9). This was to reduce possible baseline differences between the three groups. The experimenter received group allocation shortly before the intervention appointment from a not directly involved coworker.

#### 2.7.2 Data analysis and statistics

Statistical analysis was performed using R (4.1.2; R Core Team, 2021) and SPSS (IBM SPSS Statistics 24). Descriptive statistics are given as mean (standard deviation) [*M* (SD)] for continuous or number (%) [*N* (%)] for categorical variables. All tests are performed against a two-sided *α* = 0.05, and 95% confidence intervals (CI) are reported.

Previous studies showed that some participants develop intrusions after viewing a trauma film while others do not (for a meta-analysis of the trauma film paradigm see Clark et al., 2015). Studies have consistently shown that intrusions are most prevalent on the first few days after film viewing and then decline (see, e.g., Figure 2 in James et al., 2015). In order to be able to manipulate the presence of intrusions occurring after 3 days post film viewing, we were interested only in those participants who had developed intrusions after film presentation. Thus, those who scored zero in the first 3 days of the diary were excluded from further analysis (3 from the *Mobilum* group, 5 from the *Control* group). We analyzed the development of the frequency of intrusions over the six-day study period and compared the effects of the respective three conditions.

The main outcome is understood as count-based data, and intrusion rates per day were estimated by a mixed Poisson regression model. In this model, it was assumed that there was a fixed decrease in intrusion rate per day for days 1–3 (baseline phase, *t* ≤ 3).

Following reminder at day 4, an increase of the intrusion rate was considered, with different sizes due to the different interventions (*Control, Tetris, Mobilum*). Intrusion rates then were assumed to decrease again over days 4–6 (intervention phase, *t* > 3). These assumptions were based on our previous observations in a comparable study design (Kessler et al., 2020, Figure 3). A visual representation of these assumptions is shown in Supplementary Figure S1. A random slope and intercept per patient was added to the model to account for patient effects and repeated measured. This results in the following mixed Poisson model:


$$\log\left(\lambda_{\mathrm{it}}\right)=\beta_{0}+\beta_{\mathrm{time}}*t+b_{0i}+b_{1i}*t+$$

$$\begin{array}{l} \beta_{\mathrm{Control}}*\mathrm{Control}*\left(t>3\right)+\beta_{\mathrm{Tetris}}*\mathrm{Tetris}*\left(t>3\right)\\ +\beta_{\mathrm{Mobilum}}*\mathrm{Mobilum}*\left(t>3\right) \end{array}$$

In this model $\lambda_{\mathrm{it}}$ is the expected intrusion rate for participant *i* at day *t* and all β are fixed effects and $b_{0i}$ and $b_{1i}$ denotes the two correlated random effects for the intercept and slope of each participant.

*Post-hoc* comparisons between the three groups were planned in a two-staged testing protocol: In the first step, we assessed whether the *Tetris* and the *Mobilum* group differ significantly from the *Control* group in mean estimated intrusion rate over the intervention phase (*t* > 3). This is equivalent to a Dunnett test with the following hypotheses:

$H_{0}^{1}:\exp\left(\beta_{\mathrm{Tetris}}\right)/\exp\;\left(\beta_{\mathrm{Kontrolle}}\right)=1$ vs. $H_{A}^{1}:\exp\;\left(\beta_{\mathrm{Tetris}}\right)/\exp\;\left(\beta_{\;\mathrm{Kontrolle}}\right)\neq 1$ ,

and $H_{0}^{2}:\exp\left(\beta_{\mathrm{Mobile}}\right)/\exp\left(\beta_{\mathrm{Kontrolle}}\right)=1$ vs. $H_{A}^{2}:\exp\left(\beta_{\mathrm{Mobile}}\right)/\exp\left(\beta_{\;\mathrm{Kontrolle}}\right)\neq 1\text{.}$

In the second step, if both tests yield significant results, a comparison of estimated mean intrusion rate of the *Mobilum* versus the *Tetris* group with the following hypothesis would be conducted:

$H_{0}^{1}:\exp\left(\beta_{\mathrm{Mobilum}}\right)/\exp\left(\beta_{\;\mathrm{Tetris}}\right)=1$ vs. $H_{A}^{1}:\exp\left(\beta_{\mathrm{Mobilum}}\right)/\exp\left(\beta_{\mathrm{Tetris}}\right)\neq 1$ ,

For both stages, intrusion rate ratios will be reported where applicable.

## 3 Results

### 3.1 Participant characteristics, baseline data and diary compliance

A total of *N* = 112 participants were included into analysis, 68 (60.71%) of whom were female. Mean age was 23.6 years (SD 5.53). For more information, see Table 1.

**Table 1:** Baseline characteristics and diary compliance of all included participants (*N* = 112).

Measure	*M*	*SD*
ETI: Number of traumatic events	1.08	1.27
SCL-90-R: Global Severity Index	0.21	0.15
PCL-5: total score	6.08	7.52
Number of intrusions in the diary, day 1–3	11.37	8.11
Diary compliance, day 1–3	91.21	9.85
Diary compliance, day 4–6	92.63	10.63

ETI: Essen Trauma–Inventory; SCL-90-R: Symptom Checklist-90-Revised; PCL-5: PTSD Checklist for DSM-5; Number of intrusions in the diary: total count of intrusions during the first 3 days of the study; Diary compliance: self-reported diary compliance in %; M: mean; SD: standard deviation. There were no significant differences between groups for any baseline measures (tested using one-way ANOVA, significance level 5%, two-sided testing).

### 3.2 Intrusive memories of the trauma film

**Figure 3:**
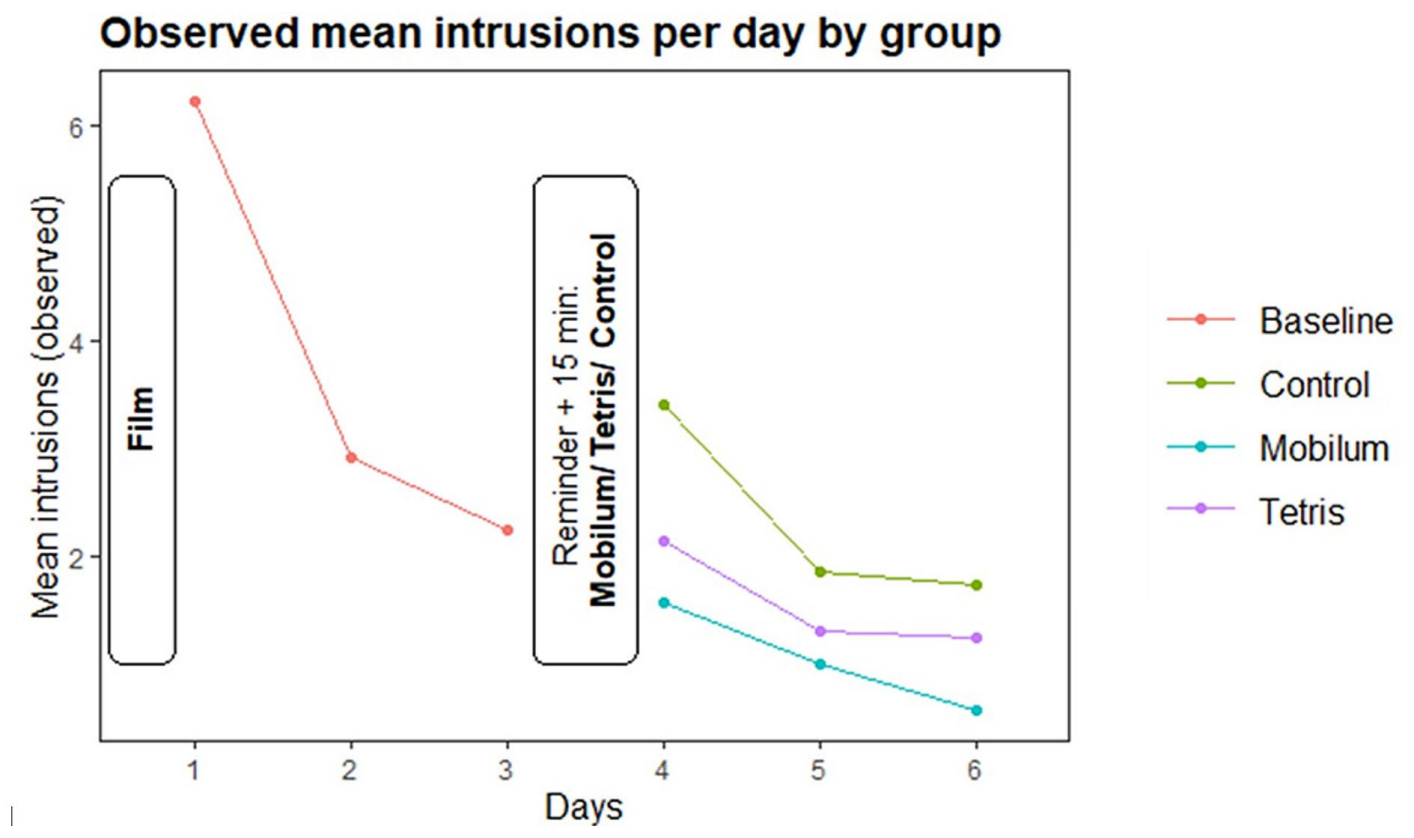
Development of mean observed intrusions per day. Red line is the combined mean for all three groups (cohort prior to randomization) during the first 3 days (pre intervention). The three colored lines represent the respective mean number of intrusions per day separated for the three intervention groups post intervention (days 4–6).

Observed mean intrusion rates in the pre-intervention phase declined as assumed through day 1–3 (Table 2; Figure 3).

**Table 2:** Observed mean intrusion rates pre-intervention (day 1–3) and post-intervention (day 4–6) in the study collective.

	Control (*N* = 35)	Tetris (*N* = 39)	Mobilum (*N* = 38)	Overall (*N* = 112)
Day 1				
Mean (SD)	6.11 (3.94)	6.38 (4.00)	6.13 (3.63)	6.21 (3.83)
Day 2				
Mean (SD)	3.17 (3.68)	2.59 (2.21)	3.03 (2.49)	2.92 (2.82)
Day 3				
Mean (SD)	2.57 (3.38)	2.08 (2.46)	2.11 (2.18)	2.24 (2.69)
Day 4				
Mean (SD)	3.40 (3.36)	2.15 (2.71)	1.58 (1.80)	2.35 (2.76)
Day 5				
Mean (SD)	1.86 (2.91)	1.31 (1.47)	1.00 (1.36)	1.37 (2.02)
Day 6				
Mean (SD)	1.74 (3.15)	1.26 (1.59)	0.58 (0.92)	1.18 (2.10)

Figure 3 shows the development of observed mean intrusions per day over the whole six-day period.

From visual inspection, the observed rates align with the assumed model (Supplementary Figure S1; Supplementary Table S1). Planned post-hoc comparisons between groups in the post intervention phase (Stage 1 of testing) are shown in Table 3.

**Table 3:** Dunnett contrasts of the mean intrusion rate ratio post intervention (*t* > 3) in the Mobilum and Tetris groups compared to the Control group (Testing stage 1).

Comparison	Intrusion rate ratio	CI	*p*-value
Post intervention: Tetris to control	0.83	0.58–1.17	0.3798
Post intervention: Mobilum to control	0.57	0.39–0.82	0.0013

The Dunnett contrasts reveal that the mean rate of intrusions post intervention in the *Mobilum* group is on average approximately 43% lower than in the *Control* group (95% CI: 17.96–61.07). The estimated mean rate of intrusions post intervention in the *Tetris* group is on average approximately 17% lower than in the *Control* group (95% CI: −17.48–41.99). Only the difference in the rate of intrusions for the *Mobilum* group is statistically significant (*p* = 0.0013).

Hence, part of our main hypothesis that the *Tetris* group would have a significantly lower intrusion rate in the 3 days after intervention compared to *Control* was not supported. The other part was supported since the *Mobilum* group had a significantly lower intrusion rate in the 3 days after intervention compared to *Control.*

According to the staged testing protocol, no subsequent comparison of the *Tetris* and *Mobilum* groups was performed.

## 4 Discussion

Expanding the time interval between (analog) trauma and administration of an intervention is crucial for future clinical purposes, since treatment of intrusions from past traumatic experiences that happened some time ago is required (Singh et al., 2020). Here, we did not replicate our previous results applying a *Tetris* intervention in a comparable study design, i.e., 3 days after a trauma film (Kessler et al., 2020). That is, contrary to our predictions, our current results do not show significantly less frequent intrusive memories by applying *Tetris* after a memory reminder cue 3 days after presentation of a trauma film compared to *Control*. On the other hand, and in line with our hypothesis, after a memory reminder the group that received the novel and bespoke visuospatial task intervention with *Mobilum* had a significant lower intrusion rate than the *Control* condition during the 3 days following the intervention. With the introduction of *Mobilum* we intended to reduce other potential aspects of the *Tetris* effect (like entertaining, exciting or distracting aspects). We consider *Mobilum* a relatively pure visuospatial task with less “gaming components” than *Tetris*. In our interpretation, our results are in line with the assumption that not only *Tetris*, but also other visuospatial tasks (in this case, *Mobilum*) might cause interference with intrusive memory processing. We conjecture that given current results, *Mobilum* may have a stronger effect than does a task such as *Tetris*, though further studies are needed.

Interestingly, there are already promising results using an intervention including *Tetris* as the visuospatial task in a form of treatment in clinical populations. In a clinical cases series, Kessler et al. (2018) examined 20 inpatients with complex posttraumatic stress disorder. The results indicated that applying *Tetris* after performing a memory reminder led to a significant reduction of intrusions of 64% compared to their own baseline level, while non-targeted intrusive memories were reduced by on average 11% (Kessler et al., 2018). In both another clinical case series and a waitlist controlled, randomized trial, an intervention including *Tetris* and a memory reminder cue also showed a reductive effect on intrusive memories (Thorarinsdottir et al., 2022; Ramineni et al., 2023). This suggests that at least under some well controlled clinical sample study conditions, an intervention procedure using *Tetris* as the visuospatial task can be beneficial. If it were the case that other tasks (such as *Mobilum*) could have stronger effects, then this warrants investigation in further clinical studies.

In contrast to the majority of studies in this research field (including our own previous Kessler et al., 2020 study), we here used a Poisson regression model for the statistical analysis. In the trauma film paradigm, daily intrusion frequency is decreasing gradually. For this reason, we consider the Poisson regression as a suitable way of modeling intrusion diary data and suggest that this could be taken into account for further research. In this context, we suggest to interpret our data with caution. At first sight, our results are likely interpreted as a reduction of intrusion frequency due to applying *Mobilum.* On the other hand, given the observed intrusion rate development day per day (see Figure 3), one alternative interpretation might say that the *Mobilum* condition only “prevents” the increase of intrusion frequency observed in the *Control* condition after the memory reminder, but beyond that has no diminishing effect on the natural course of intrusions stemming from the trauma film 3 days prior. In the light of previous research, we consider that to be unlikely, but cannot rule that possibility out, because we had no condition with the absence of both the memory reminder and an intervention. Hence, we have no natural development of intrusion frequency in this paradigm as a benchmark and see a relevant limitation in this.

There are various other limitations of the current study. First, we had no non-visuospatial condition, so we cannot rule out that the effect of *Mobilum* could have been obtained with a task that has for example comparable demands on attention and concentration, but requires no visuospatial capacities. Second, we investigated an analog trauma (a film). Hence, the transfer to clinical populations should be considered with caution. Third, intrusion and compliance measurement was based on a self-report system and objective measures could be investigated in the future. Fourth, there were no restrictions to (and no assessment of) participants’ activities outside the lab during the observation period. Possible confounding factors during this time (e.g., watching horror movies with disturbing visual content) were therefore not assessed.

With the introduction of *Mobilum* as a new task within an intervention to reduce intrusion rate after a trauma film, we see the opportunity to investigate the visuospatial approach more precisely. In *Mobilum* many variables can be fully controlled by the researcher which may potentially modulate the effect of the intervention or its feasibility. Furthermore, there is no risk due to potential commercial aspects (as possibly in the case of *Tetris*). Even though it is highly hypothetical at this point of research, it might seem plausible that *Mobilum* has an even stronger effect than *Tetris* following the concepts of working memory modularity and dual-task interference. Interestingly, we would expect the observed order of effects: *Control* < *Tetris* < *Mobilum*. Earlier studies applying *Tetris* may have overestimated the effect size especially when delivered 3 days after the experimental trauma exposures since most are given at shorter time intervals. In other words, our study may have been underpowered with regard to the *Tetris* effect.

Further research should compare different types of interventions and, for the translational aspect, should try to investigate more realistic contexts. With regard to the mechanistic hypothesis that it is a visuospatial task *per se* interfering with intrusive memory processing, *Mobilum* could be tested against another task which is cognitively demanding but considered as less or non-visuospatial. For *Tetris*, such studies have already been conducted, comparing it to verbal quiz games (Holmes et al., 2010; Kessler et al., 2020) or another verbal task (“word games”; Hagenaars et al., 2017) in healthy subjects using the trauma film paradigm, and comparing it to reading a Wikipedia article in a cross-over RCT in PTSD patients (Kehyayan et al., 2024), however with diverging, overall inconclusive results. In this context, we also see the open question if at least to some degree visuospatial working memory is always involved solving complex cognitive tasks. Then, a task would be considered more or less visuospatially demanding rather than categorized into visuospatial or non-visuospatial.

Bridging basic research to the clinic, the adjusted trauma film paradigm of Hilberdink and coworkers could be one step in the right direction closing the gap between laboratory and clinical application. Combining the trauma film with the socially-evaluated cold pressor test led to more intrusions and an enhanced stress reaction compared to applying the trauma film alone (Hilberdink et al., 2022). The authors interpreted their analog trauma as likely more comparable to a real-life trauma. Results might be more transferable to clinical populations. Another possibility is to investigate *Mobilum* directly in clinical populations. One option might be using a case series design such as Thorarinsdottir and colleagues and also test its acceptability to patients (Thorarinsdottir et al., 2022).

In summary, our results support the idea that a traumatic memory trace that already has been consolidated (i.e., 3 days after experimental trauma), at least under certain conditions can be modified by using the combination of a memory reminder and a visuospatial task. We showed that a visuospatial computer game play task *Mobilum* (i.e., one which is not a commercial computer game) may reduce intrusions after analog trauma. There may be advantages of further investigating *Mobilum,* even though there are some promising results using the other game *Tetris* in clinical populations (e.g., Kessler et al., 2018; Horsch et al., 2017; Iyadurai et al., 2018). This line of experimental investigation is important since imagery-competing task interventions would have two major advantages over existing treatments post-trauma. First, they would be simple to apply, which means they could ideally be delivered without professional help. Second, they would be easily accessible, since the procedure is relatively independent from language skills (the reactivation can be done in native language) and thus be cost-efficient. Building on these advantages, those visuospatial interventions could be primarily used to augment a specialized psychotherapy or for bridging the time period until such a therapy is available. On a global scale, this type of intervention may in the future even be used as a first line approach as long as other established forms of treatment are not available. However, as already mentioned above, the study presented here uses the trauma film as an “analog trauma” to investigate intrusion development in a healthy study population. Results need to interpreted against this background, as it is not possible to directly transfer the obtained results to populations of traumatized patients. Therefore, further clinical research is warranted to show whether (and under which conditions) the promising results from fundamental research can be translated to trauma patients actually suffering from intrusive memories.

## Data Availability

The datasets presented in this article are not readily available because no additional data are available. The participants of this study did not give written consent for their data to be shared publicly, so due to the sensitive nature of the research, individual participant data is not available. Requests to access the datasets should be directed to aram.kehyayan@rub.de.
