# Supplementary material for: Comparing a new visuospatial intervention administered 3 days after a trauma film to reduce the occurrence of intrusive visual memories: a single-center randomized, controlled trial in healthy participants
Source: Front Psychol. 2025 Jan 10;15:1454086. doi: 10.3389/fpsyg.2024.1454086 (PMC11759303; doi:10.3389/fpsyg.2024.1454086)
Supplement: Supplementary file 1 [file Data_Sheet_1.docx]

**Supplementary Figure 1:** *Graphical representation of the assumed course of the mean log intrusion rate. On day 1, a film is presented to the total study collective, inducing intrusions. Number of intrusions per participant are noted for day 1 through 3 (“baseline”) via diary entries. We assume a linear decline in intrusion rate in the baseline period, as shown by the solid green line. On day 4, participants are randomized to either the “Tetris”, “Mobilum” or “Control” conditions and shown a reminder film and administered the intervention. We hypothesize a further linear decline in intrusion rates, were no reminder given (dashed green line). With the reminder present, we hypothesize an increase in intrusions in all 3 conditions on day 4 compared to the linear decline where no reminder given (ßk). However, we assume both active conditions (“Tetris” and “Mobilum”) to have a less pronounced increase compared to the “Control” condition. These assumptions are modeled in the GLMM Poisson described in the statistical methods section.*
