# Supplementary material for: Comparing a new visuospatial intervention administered 3 days after a trauma film to reduce the occurrence of intrusive visual memories: a single-center randomized, controlled trial in healthy participants
Source: Front Psychol. 2025 Jan 10;15:1454086. doi: 10.3389/fpsyg.2024.1454086 (PMC11759303; doi:10.3389/fpsyg.2024.1454086)
Supplement: Supplementary file 2 [file Table_1.docx]

| **Supplementary Table 1:** *Results of the mixed Poisson regression model used to estimate the intrusion rates per day, study phase and intervention group.* | | | |
| --- | --- | --- | --- |
|  |  | | |
| *Predictors* | *Intrusion Rate Ratios* | *CI* | *p-value* |
| (Intercept) | 9.185 | 7.901 - 10.678 | **<0.001** |
| Day | 0.557 | 0.519 - 0.598 | **<0.001** |
| Group [Control] * Phase | 2.296 | 1.762 - 2.992 | **<0.001** |
| Group [Tetris] * Phase | 1.896 | 1.443 - 2.491 | **<0.001** |
| Group [Mobilum] * Phase | 1.298 | 0.966 - 1.743 | 0.083 |

*Supplementary Table 1 shows the estimated intrusion rate ratios for day of the study and the intervention group by study phase (baseline vs intervention phase) interaction. We found a significant effect of time (day) on the overall intrusion rate, with mean intrusion rates decreasing each day. Moreover, a statistically significant interaction of the Control and Tetris groups, but not the Mobilum group, with the study phase was found. These results are concordant with the observed mean intrusion rates (Figure 3), where a pronounced increase in mean intrusion rate on day 4 compared to day 3 is visible for the Control and Tetris groups, but not the Mobilum group. Estimated rates then where tested as described in the methods section.*
