# Supplementary figures and images for: Comparing a new visuospatial intervention administered 3 days after a trauma film to reduce the occurrence of intrusive visual memories: a single-center randomized, controlled trial in healthy participants
Source: Front Psychol. 2025 Jan 10;15:1454086. doi: 10.3389/fpsyg.2024.1454086 (PMC11759303; doi:10.3389/fpsyg.2024.1454086)

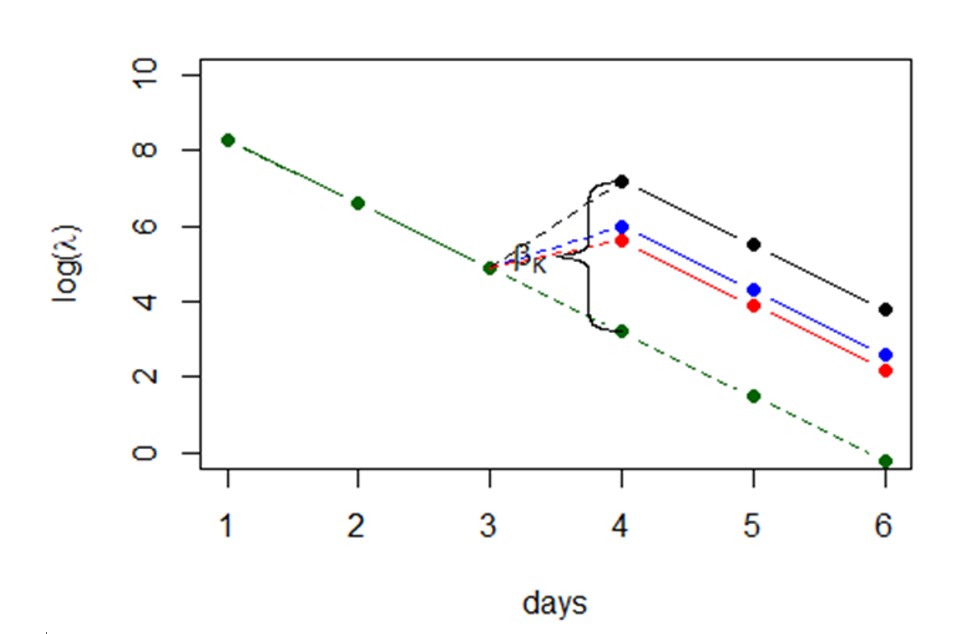

Supplement: Supplementary file 3 [file Image_1.jpeg]
